# Soluble fibrinogen-like protein 2 levels in patients with hepatitis B virus-related liver diseases

**DOI:** 10.1186/s12879-018-3473-2

**Published:** 2018-11-12

**Authors:** Hoang Van Tong, Nguyen Van Ba, Nghiem Xuan Hoan, Mai Thanh Binh, Dao Thanh Quyen, Ho Anh Son, Hoang Van Luong, Do Quyet, Christian G. Meyer, Le Huu Song, Nguyen Linh Toan, Thirumalaisamy P. Velavan

**Affiliations:** 1 Institute of Biomedicine and Pharmacy, Vietnam Military Medical University, 222 Phung Hung, Ha Dong, Hanoi, Vietnam; 2 Department of Pathophysiology, Vietnam Military Medical University, Hanoi, Vietnam; 3 Institute of Tropical Medicine, University of Tübingen, Wilhelmstrasse 27, 72074 Tübingen, Germany; 4 108 Military Central Hospital, Hanoi, Vietnam; 5 Vietnamese-German Center of Excellence in Medical Research, Hanoi, Vietnam; 6 Medical Faculty, Duy Tan University, Da Nang, Vietnam

**Keywords:** sFGL2 levels, HBV infection, Viral hepatitis, Liver cirrhosis, Hepatocellular carcinoma

## Abstract

**Background:**

Clinical progression of HBV-related liver diseases is largely associated with the activity of HBV-specific T cells. Soluble fibrinogen-like protein 2 (sFGL2), mainly secreted by T cells, is an important effector molecule of the immune system.

**Methods:**

sFGL2 levels were determined by ELISA assays in sera of 296 HBV patients clinically classified into the subgroups of acute hepatitis B (AHB), chronic hepatitis B (CHB), liver cirrhosis (LC), hepatocellular carcinoma (HCC) and patients with LC plus HCC. As control group, 158 healthy individuals were included. *FGL2* mRNA was quantified by qRT-PCR in 32 pairs of tumor and adjacent non-tumor liver tissues.

**Results:**

sFGL2 levels were elevated in HBV patients compared to healthy controls (*P* <  0.0001). In the patient group, sFGL2 levels were increased in AHB compared to CHB patients (*P* = 0.017). sFGL2 levels were higher in LC patients compared to those without LC (*P* = 0.006) and were increased according to the development of cirrhosis as staged by Child-Pugh scores (*P* = 0.024). Similarly, HCC patients had increased sFGL2 levels compared to CHB patients (*P* = 0.033) and *FGL2* mRNA was up-regulated in tumor tissues compared to adjacent non-tumor tissues (*P* = 0.043). In addition, sFGL2 levels were positively correlated with HBV-DNA loads and AST (Spearman’s rho = 0.21, 0.25 and *P* = 0.006, 0.023, respectively), but reversely correlated with platelet counts and albumin levels (Spearman’s rho = − 0.27, − 0.24 and *P* = 0.014, 0.033, respectively).

**Conclusions:**

sFGL2 levels are induced by HBV infection and correlated with the progression and clinical outcome of HBV-related liver diseases. Thus, sFGL2 may serve as a potential indicator for HBV-related liver diseases.

## Background

Hepatitis B virus (HBV) infection is a major health problem with approximately 257 million people infected and 887,000 deaths annually due to complications [1]. HBV is highly prevalent in sub-Saharan Africa, Asia and parts of America with infection rates ranging from 8 to 20% of the populations [1]. Vietnam is a highly endemic country for HBV infection with a prevalence of up to 20%. In spite of the effective coverage of anti-HBV vaccination in Vietnam, HBV-related liver diseases are foreseen to be an important public health problem in the next decades due to the long latency of chronic hepatitis, liver cirrhosis, and hepatocellular carcinoma [2]. Estimations made in 2012 have indicated that up to 10 million people are living with chronic hepatitis B in Vietnam with 23,300 deaths annually due to the infection [3].

HBV infection leads to a wide spectrum of liver diseases, including an asymptomatic carrier status, acute self-limiting and fulminant hepatitis, chronic hepatitis B (CHB), liver cirrhosis (LC) and hepatocellular carcinoma (HCC) [1]. CHB is the most important risk factor for the development of HCC with a 100-fold increase in chronic HBV carriers compared to non-carriers [4]. HCC is the third leading cause of cancer-related deaths and more than 500,000 new cases are diagnosed worldwide annually [4]. HBV stimulates both the innate and adaptive immune systems to establish persistent infections and the liver injuries in chronic infection are associated with the activity of HBV-specific T cells [5]. The mechanism of hepatocellular injury is immune-mediated and strongly dependent on the interaction between distinct viral factors and host immune responses [5]. Sequential transformation of normal hepatocytes to malignant cells during HCC development is associated with the immune response to HBV-infected liver cells, accumulated genetic alterations, and the interaction between the viral HBx protein with host signaling proteins [6].

Fibrinogen-like protein 2 (FGL2), also known as fibroleukin, belongs to the fibrinogen-associated superfamily of proteins and is homologous to the β and ɤ chains of fibrinogen [7]. FGL2 is encoded by the *FGL2* gene, which contains two exons and is localized to the proximal region of chromosome 7q11.23 (NC_000007.14) [8]. There are two different forms of FGL2, namely the type II transmembrane FGL2 (mFGL2) and the soluble FGL2 (sFGL2). mFGL2 has prothrombinase activity to cleave thrombin from prothrombin and is expressed on the surface of different cell types such as macrophages, endothelial and dendritic cells [8, 9], while sFGL2 is mainly secreted by CD4+, CD8+ and regulatory T cells, and has immune regulatory activity [10, 11]. sFGL2 is an important effector molecule involved in various processes of immunity, including antigen presentation, immunosuppression and apoptosis [7]. It is also part of various signaling pathways such as ITAM/ITIM (immunoreceptor tyrosine-based activating motif/ immunoreceptor tyrosine-based inhibitory motif), NF-κB (nuclear factor kappa-light-chain-enhancer of activated B cells) and MAPK (mitogen-activated protein kinases) [7, 12].

Clinically, sFGL2 plays an important role in organ transplantation through regulation of T and B cell mediated immunity. Increased sFGL2 levels have been observed in recipients with acute allograft rejection [12, 13]. sFGL2 has been implicated in different types of diseases, including cancer, autoimmune and infectious diseases [7, 14–16]. In viral hepatitis, sFGL2 is involved in the immune responses against HBV and HCV infections. Expression of FGL2 was associated with susceptibility to murine hepatitis virus strain 3 (MHV-3) infection in vivo [11], and the *FGL2* gene has been suggested as a potential target for treatment of fulminant viral hepatitis [11, 17, 18]. In a clinical study, plasma sFGL2 levels were significantly increased and correlated with clinical progression of HCV infection and antiviral therapy [19]. In addition, sFGL2 regulates the T-cell function in cirrhotic patients with HCC [14], and plasma sFGL2 levels are positively associated with the severity of non-alcoholic fatty liver disease (NAFLD) [16]. The present study investigates plasma levels of sFGL2 in Vietnamese patients with HBV-related liver diseases and their correlation with clinical progression of HBV infection.

## Methods

### Patients and controls

Two hundred and ninety-six Vietnamese patients (*n* = 296) with HBV infections were recruited from the 108 Military Central Hospital and 103 Military Hospital, Hanoi, Vietnam between the years 2014 and 2016 [20]. All recruited HBV patients were negative for HCV and HIV. The recruited chronic HBV patients were diagnosed based on the guidelines of European Association for the Study of the Liver (EASL) [21], and the HCC patients based on the guidelines from American Association for the Study of Liver Diseases (AASLD) [22]. The patients were further sub-classified into five groups based on the clinical manifestations, biochemical and serological parameters. The patient groups were acute hepatitis B (AHB; n = 29), chronic hepatitis B (CHB; *n* = 73), patients with only liver cirrhosis (LC; *n* = 70), patients with only hepatocellular carcinoma (HCC; *n* = 99) and patients with both liver cirrhosis and hepatocellular carcinoma (HCC + LC, *n* = 25). In addition, patients with LC were further classified either as Child-A, Child-B or Child-C according to Child-Pugh scores [23]. Laboratory and clinical parameters such as blood counts, total and direct bilirubin, prothrombin, albumin, alanine transaminase (ALT), aspartate transaminase (AST), alpha-fetoprotein (AFP) and HBV-DNA loads were measured by routine laboratory tests. As control group, we recruited one hundred and fifty-eight healthy Vietnamese blood donors (HC; *n* = 158) who were confirmed negative for HBsAg, anti-HCV and anti-HIV antibodies [20]. All control individuals had no history of alcohol or drug use. Approximately five milliliters of venous blood was collected and respective serum and/or plasma was separated from whole blood immediately and stored at − 20 °C until further use. In addition, 32 pairs of tumor and adjacent non-tumor tissues were collected from HCC patients who underwent surgery at the 108 Military Central Hospital. The clinical profiles of the HCC patients have been described in our previous published study [20] [24].

### Ethics approval and consent to participate

Informed written consent was received from all participants after detailed explanation of the study at the time of blood sampling. The study was approved by the Institutional Review Board of the Vietnam Military Medical University (VMMU) and the 108 Military Central Hospital, Hanoi, Vietnam. All experiments were performed in accordance with relevant guidelines and regulations.

### Quantification of sFGL2 levels by ELISA

Soluble FGL2 levels were measured in the plasma samples from the patients with HBV-related liver diseases and in healthy controls using a commercially available Fibrinogen-Like 2 (FGL2) ELISA kit (Wuhan USCN Business Co., Ltd., Wuhan, China) following the manufacturer’s instructions. The microtiter plate provided of the kit was delivered already coated with a specific FGL2 monoclonal antibody. 100 μL of the FGL2 standard and the study samples were added to the wells of the coated ELISA plate and incubated for 2 h at 37 °C. The liquid of each well was removed and 100 μL of the first detection solution with a biotin-conjugated antibody preparation specific for FGL2 was added and incubated for 1 h at 37 °C. After washing with wash solution, the second detection solution with avidin conjugated to Horseradish Peroxidase (HRP) was added to each microplate well and incubated for 30 min at 37 °C. After washing again, TMB substrate solution was added and incubated for 15–25 min at 37 °C. Subsequently, stop solution was added and the plates were immediately read at a wavelength of 450 nm by an ELISA reader. The standard curve was plotted based upon the mean of O.D. (optical density) value and the known concentration of the standard. The concentrations of sFGL2 protein were calculated based upon the standard curve. The minimum detectable limit of sFGL2 proteins was less than 0.19 ng/mL.

### Quantification of *FGL2* mRNA by RT-PCR

Total RNA was extracted from the 32 tumour and adjacent non-tumour tissues using Trizol reagent (Life Technologies, Carlsbad, CA, USA) and was reverse transcribed into cDNA by using QuantiTect Reverse Transcription Kit (Qiagen, Hilden, Germany) [20]. Quantification of cDNA was performed by quantitative real-time PCR with *GAPDH* (glyceraldehyde-3-phosphate dehydrogenase) as a reference gene. Primer sequences were FGL2_F: 5′-AGG CAG AAA CGG ACT GTT GT-3′ and FGL2_R: 5’-CCA GGC GAC CAT GAA GTA CA-3′, GAPDH_F: 5’-TGC ACC ACC AAC TGC TTA GC-3′ and GAPDH_R: 5’-GGC ATG GAC TGT GGT CAT GAG-3′ [25]. In brief: real-time PCR amplification was carried out in a reaction volume of 25 μl containing 12.5 μl 2X SYBR Green PCR master mix (Bioline, Luckenwalde, Germany), 0.5 μM specific primer pairs for target gene or reference gene, 5 ng cDNA and RNase-free water up to 25 μl of reaction volume. Thermal conditions were initial denaturation at 95 °C for 2 min followed by 45 cycles of denaturation at 95 °C for 5 s, primer specific annealing and an extension step at 58 °C for 20 s. Melting curve analyses starting from 58 °C to 85 °C were performed after each run to confirm the presence of specific PCR products [20]. All reactions were performed in duplicates and each run was repeated twice using the LightCycler® 480 real-time PCR system (Roche, Basel, Switzerland). The relative expression of *FGL2* mRNA was calculated based on the ΔCt algorithm and by normalizing the expression of the house keeping gene *GAPDH*.

### Statistical analysis

Quantitative variables were tested for normality and were presented as mean and standard deviation if the data are normally distributed. Normally distributed data were compared using Student’s t-test and ANOVA for two and/or more than two groups, respectively. If the data are not normally distributed, quantitative variables were presented as medians with range and were compared using Mann Whitney Wilcoxon and Kruskal-Wallis test for two and more than two groups, respectively. Parametric Pearson’s correlation coefficient or non-parametric Spearman’s rank correlation coefficient were used to correlate the given two variables, where appropriate. Paired-samples t test was used to compare the relative expression of *FGL2* mRNA between tumour and adjacent non-tumour tissues. The SPSS software (SPSS Statistics, IBM, Armonk, NY, the USA) was used for all statistical analyses and the significant level was set at *P <* 0.05.

## Results

### Baseline characteristics of study participants

The demographic characteristics such as age, gender and clinical parameters such as blood counts, liver function tests, HBV-DNA load and the tumor marker alpha-feto protein (AFP) of all investigated Vietnamese hepatitis B patients and healthy controls are presented in Table 1. Red blood cell counts was observed to be lowest among patients with HCC followed by patients with LC + HCC, LC and CHB patients (*P* <  0.01). White blood cell counts were lower in patients with LC and patients with LC plus HCC compared to CHB and HCC patients (*P* <  0.001). Platelet counts was also observed to be lowest among LC patients followed by LC + HCC, CHB and HCC patients (*P* <  0.001). The levels of ALT, AST, total and direct bilirubin were significantly higher in the AHB group compared to other patient groups (Table 1). In chronically affected patients, the liver enzyme levels such as ALT, AST and albumin levels were higher compared to those with advanced liver diseases (LC, HCC) (*P* <  0.001). Also that total and direct bilirubin levels was higher, whereas albumin and prothrombin levels were lower in LC patients compared to the other groups (*P* <  0.001). As expected, the level of alpha-feto protein levels was observed to be elevated in patients with HCC than in patients without HCC (*P* <  0.001) (Table 1).

**Table 1:** Characteristics of patients with HBV-related liver disease segregated according to clinical status

Characteristics	AHB (*n* = 29)	CHB (*n* = 73)	LC (*n* = 70)	HCC (*n* = 99)	LC + HCC (*n* = 25)	HC (*n* = 158)	*P* value
Age (median, range)	32 (17–70)	39.5 (20–76)	48 (17–74)	50 (15–79)	56 (37–81)	31 (19–38)	< 0.001
Gender (male/female)	23/6	55/18	54/16	84/15	25/0	112/46	< 0.001
Liver cirrhosis stage:							
*Child-Pugh A (n,%)*	NA	NA	22 (49%)^a^	NA	15 (60%)	NA	NA
*Child-Pugh B (n,%)*	NA	NA	12 (28%)^a^	NA	8 (32%)	NA	NA
*Child-Pugh C (n,%)*	NA	NA	10 (23%)^a^	NA	2 (8%)	NA	NA
RBC (×10^3^/ml)	NA	6.7 (4.5–13.9)	6 (2.7–20.5)	5.8 (4–11)	5.7 (2.7–11)	NA	< 0.01
WBC (× 10^6^/ml)	NA	4.9 (4.1–5.3)	4.3 (2.3–6.7)	4.9 (4.2–6)	4.5 (3–6)	NA	< 0.001
PLT (×10^3^/ml)	NA	168.5 (61–355)	87.5 (49–299)	196 (101–361)	122 (35–237)	NA	< 0.001
AST (IU/L)	1109 (115.5–4593)	143 (77–657)	71 (16.8–1059)	67 (14–371)	63 (25–655)	NA	< 0.001
ALT (IU/L)	861.5 (182–4425)	144 (89–1643)	57 (8–1426)	50.5 (3–471)	54 (22–551)	NA	< 0.001
Total-Bilirubin (mg/dl)	132 (21.8–558)	22.5 (12–412)	30.3 (6.5–722)	19 (5.1–282)	23 (9–185)	NA	< 0.001
Direct-Bilirubin (mg/dl)	117.3 (14.3–512)	12.4 (6.2–292.3)	14 (1.3–450)	8.8 (2–189.4)	8 (2–59)	NA	< 0.001
Albumin (g/L)	NA	42 (33–50)	32.5 (25–39)	39 (30–47)	38 (27–44)	NA	< 0.01
Prothrombin (%)	85 (50–120)	76 (26–122)	46 (15–100)	78 (37–100)	77 (19.6–117)	NA	< 0.01
HBV-DNA (log10 copies/ml)	3.96 (3.5–5.7)	4.2 (2.8–4.66)	4.1 (2.8–6.4)	3.98 (2.5–8.9)	6 (2.3–10.4)	NA	< 0.001
Alpha fetoprotein (IU/L)	NA	12.5 (2–151)	6.7 (1.2–1050)	96.7 (1.5–1050)	113 (2–300)	NA	< 0.001

Abbreviations: *AHB* acute hepatitis B, *CHB* chronic hepatitis B, *LC* liver cirrhosis, *HCC* hepatocellular carcinoma, *LC + HCC* patients with both LC and HCC, *HC* healthy control, *RBC* red blood cells, *WBC* white blood cells, *PLT* platelets, *AST and ALT* aspartate and alanine amino transferase, *AFP* alpha-fetoprotein, *IU* international unit, *NA* not applicable. Values given are medians and ranges. *P* values were calculated by Chi-squared test and Kruskal-Wallis test where appropriate. ^a^Only patients with clear classification and Child-Pugh score available

### Soluble FGL2 levels in patients with HBV-related liver diseases

**Fig. 1:**
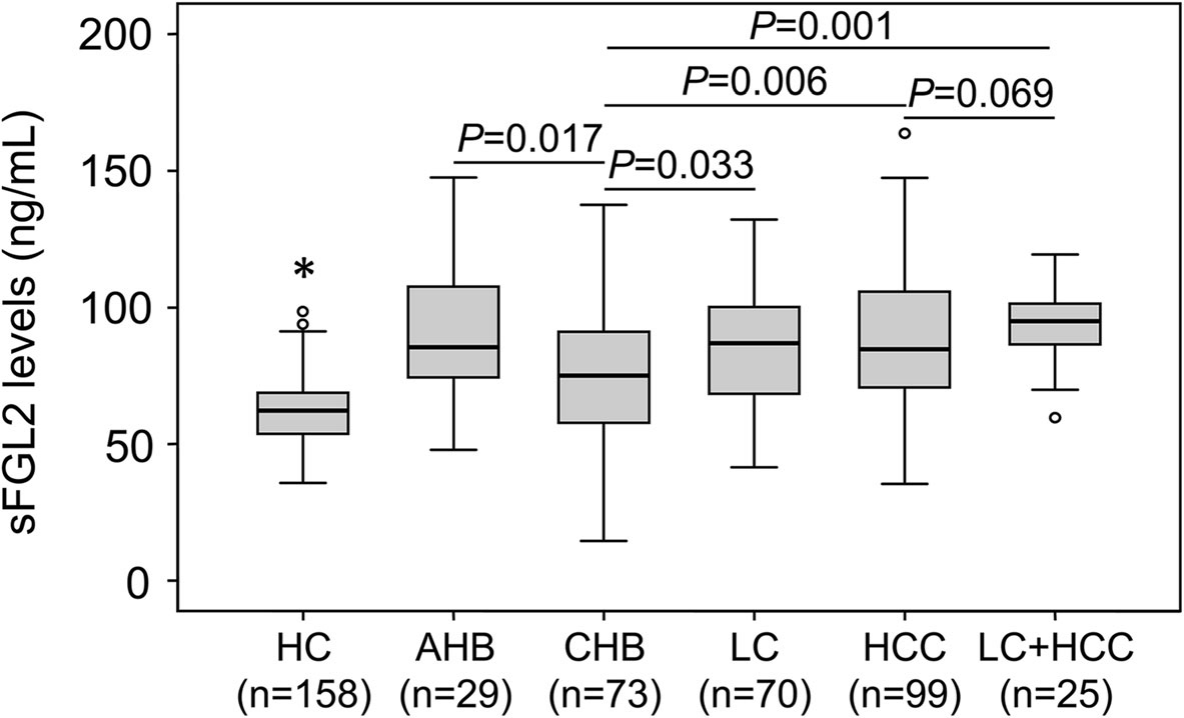
sFGL2 levels in patients with HBV-related liver diseases and in healthy controls. sFGL2 levels were measured in study subjects and compared between subgroups. HC, healthy controls; AHB, acute hepatitis B; CHB, chronic hepatitis B; LC, patients with liver cirrhosis; HCC, patients with hepatocellular carcinoma; LC + HCC, patients with both liver cirrhosis and hepatocellular carcinoma. (*): *P* < 0.0001 for comparison with other groups. Box plots illustrate medians with inter-quartile range. *P* values were calculated by Mann-Whitney-Wilcoxon test

Soluble FGL2 levels were measured in different clinical subgroups of patients with HBV-related liver diseases and in healthy controls. We observed a mean of 91.1 ± 26.6 ng/ml in AHB, 77.2 ± 25.7 ng/ml in CHB, 85.2 ± 22.7 ng/ml in LC, 87.9 ± 24.8 ng/ml in HCC patients and 92.8 ± 14.1 ng/ml in LC + HCC patients. In healthy individuals, the mean was 62.4 ± 11.9 ng/ml. The results clearly show that sFGL2 levels were significantly elevated in patients with HBV-related liver diseases compared to controls (*P* <  0.0001) (Fig. 1). In the patient group, sFGL2 levels were increased in AHB compared to CHB patients (*P* = 0.017). The results indicate that sFGL2 levels are modulated according to the stage of HBV infection. Increased sFGL2 levels were observed in patients with advanced liver diseases such as LC, HCC patients and those with both LC and HCC compared to CHB patients (*P* = 0.033, 0.006, and 0.001, respectively). There was no difference of sFGL2 levels in comparisons of LC with HCC patients (*P* > 0.05). Nevertheless, sFGL2 levels were higher in patients with both LC and HCC compared to those with LC or HCC alone, but the difference did not reach significance (Fig. 1).

**Fig. 2:**
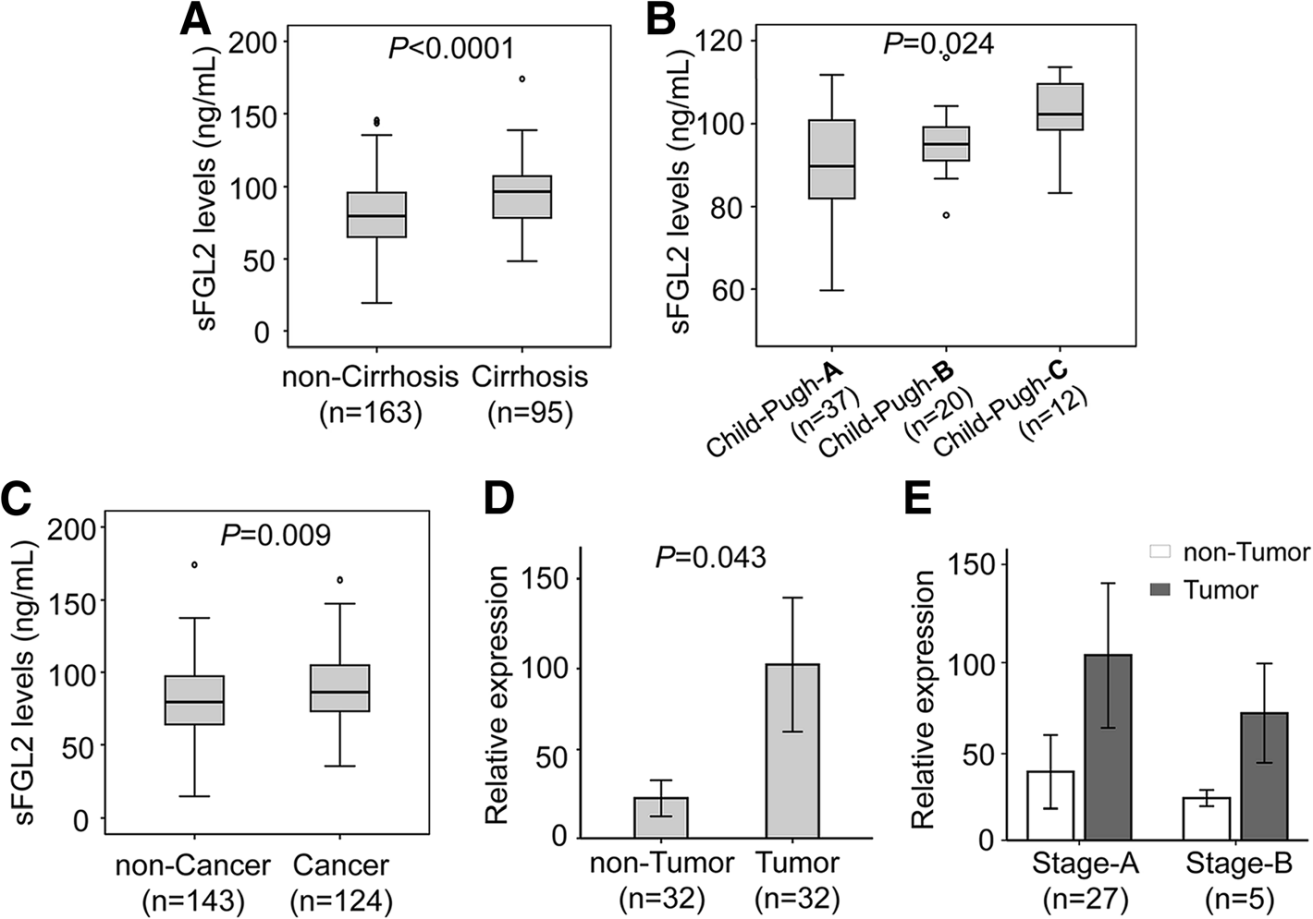
sFGL2 levels in patients with HBV-related liver diseases and in healthy controls. sFGL2 levels were measured in study subjects and compared between subgroups. **a**: comparison between chronic patients with and without liver cirrhosis. **b**: comparison among different Child-Pugh groups of patients with liver cirrhosis. **c**: comparison between chronic patients with and without hepatocellular carcinoma. **d**: comparison of *FGL2* mRNA expression in tumour and adjacent non-tumour tissues. **e**: *FGL2* mRNA expression in stage-A and stage-B tumour and adjacent non-tumour tissues. Box plots illustrate medians with inter-quartile range. Comparisons of sFGL2 levels were performed using Mann-Whitney-Wilcoxon test or Kruskal-Wallis test, while comparisons of *FGL2* mRNA relative expression were performed using Paired-samples t test

In the patient group with chronic HBV-related liver diseases, we stratified the patients into subgroups with and without LC, and those with and without HCC. sFGL2 levels were significantly increased in the patients with LC compared to those without LC (*P* <  0.0001) (Fig. 2a). Patients with LC were further categorized as Child-Pugh-A, Child-Pugh-B and Child-Pugh-C based on Child-Pugh scores if available. Child-Pugh-C LC patients had higher sFGL2 levels, followed by Child-Pugh-B and Child-Pugh-A LC patients (*P* = 0.024) (Fig. 2b). Similarly, patients with HCC had significantly higher sFGL2 levels compared to the patients without HCC (*P* = 0.009) (Fig. 2c). Furthermore, we examined the expression of *FGL2* mRNA in tumor and adjacent non-tumor tissues. The relative expression of *FGL2* mRNA was significantly up-regulated in tumor tissues compared to adjacent non-tumor tissues (*P* = 0.043) (Fig. 2d). We then examined whether *FGL2* mRNA relative expression was associated with the development of HCC by analyzing *FGL2* mRNA relative expression according to the BCLC staging classification. However, *FGL2* mRNA relative expression did not differ between stage A and B HCC tissues as well as between the corresponding adjacent non-tumor tissues obtained from the same stage A and B HCC patients (*P* > 0.05) (Fig. 2e). These results indicate that sFGL2 levels are associated with advanced HBV-related liver diseases.

### Correlation between sFGL2 levels and clinical parameters

**Fig. 3:**
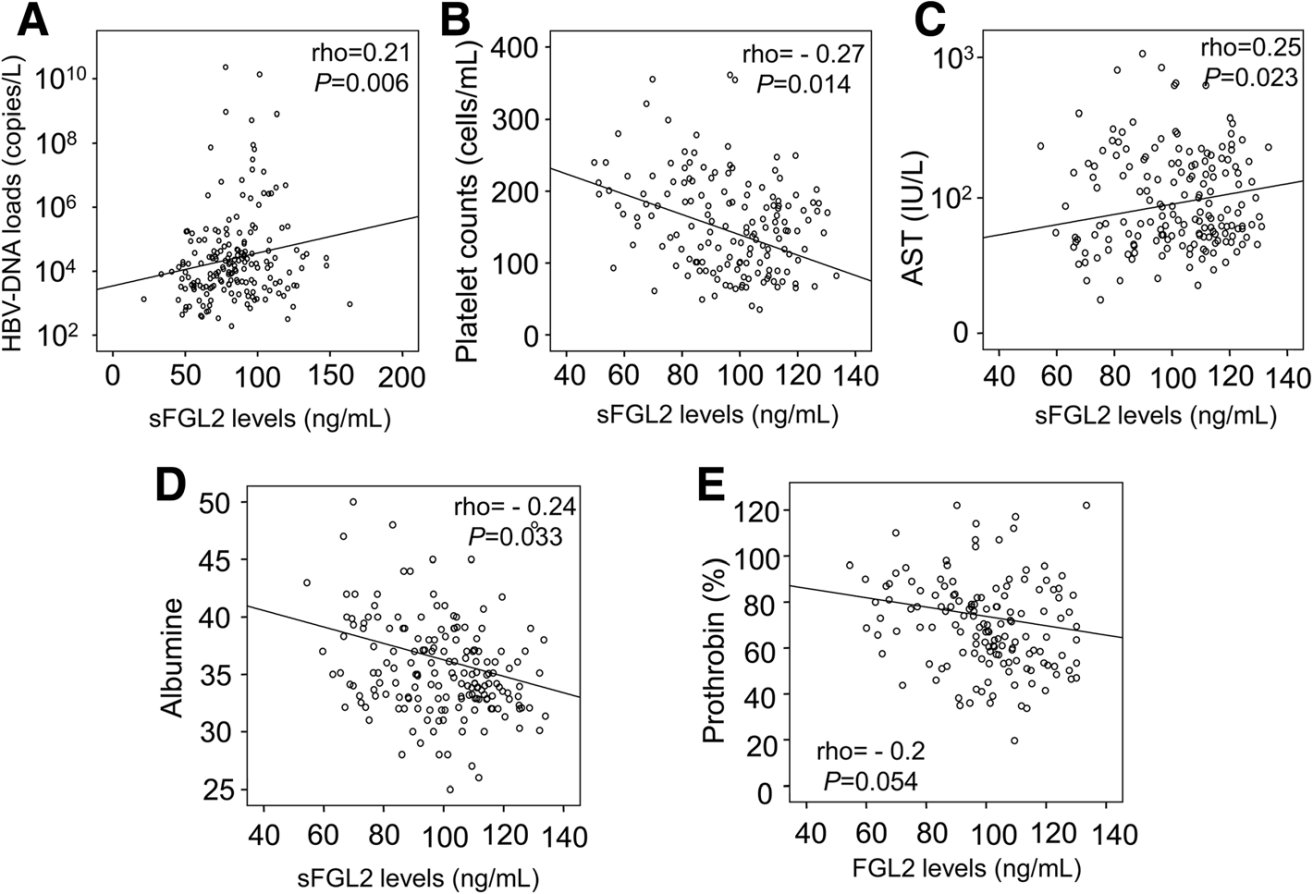
Correlation of sFGL2 levels with clinical parameters of HBV infection. Correlations of FGL2 levels with different available clinical parameters were calculated by using Spearman’s rank correlation coefficient test. The Spearman’s rho and *P* value are also presented. **a**: between sFGL2 levels and HBV-DNA loads; **b**: between sFGL2 levels and platelet counts; **c**: between sFGL2 levels and aspartate amino transferase (AST) levels; **d**: between sFGL2 levels and albumin levels; and **e**: between sFGL2 levels and prothrombin

We analyzed the correlations of sFGL2 levels with available clinical and laboratory parameters of HBV infection in HBV patients and observed a significant positive correlation of sFGL2 levels with HBV-DNA loads and with AST levels (Spearman’s rho = 0.21, *P* = 0.006, and Spearman’s rho = 0.25, *P* = 0.023, respectively) (Fig. 3). sFGL2 levels were significantly and reversely correlated with platelet counts and albumin levels (Spearman’s rho = − 0.27, *P* = 0.014, and Spearman’s rho = − 0.24, *P* = 0.033, respectively) (Fig. 3). However, sFGL2 levels were either not or weakly only correlated with the levels of total and direct bilirubin, ALT and prothrombin.

## Discussion

sFGL2 is a regulatory molecule of the immune system and involved in the pathogenesis of many diseases, including viral hepatitis [7, 14–16]. In this study, we have shown that sFGL2 levels are significantly altered in HBV-related liver diseases compared to healthy controls and are highest among patients with acute hepatitis B. sFGL2 levels are increased according to the clinical progression of chronic HBV-related liver diseases and are correlated with several clinical parameters such as AST, albumin, platelet counts and HBV-DNA loads. sFGL2 appears to play an important role in the clinical outcome of HBV infection and the progression of HBV-related liver diseases.

The findings that sFGL2 levels are increased according to the progression of chronic HBV-related liver diseases are in accordance with previous studies [14, 16, 19]. The observation that sFGL2 levels are strongly elevated in acute hepatitis B patients compared to those in CHB supports previous in vivo studies in mice showing that infection with MHV3 induces FGL2 expression [11, 26]. Induction of FGL2 during viral infection has been established in animal models, showing that plasma FGL2 levels are considerably elevated 2 days after infection with lymphocytic choriomeningitis virus strain WE (LCMV-WE) [18]. In HCV infection, plasma FGL2 levels are extensively increased in patients with chronic hepatitis compared to healthy controls [19]. The increasing levels of sFGL2 in the acute phase of hepatitis in our study group supports the previous finding that FGL2 is involved in the pathogenesis and clinical outcome of fulminant hepatitis in animal models [27, 28].

Plasma FGL2 levels are higher in patients with HCV-related LC compared to those without cirrhosis and correlated with more severe cirrhosis [19]. Similarly, our results also indicate that sFGL2 levels are elevated in patients with HBV-related cirrhosis compared to those without cirrhosis, and sFGL2 levels are increased according to the stage of cirrhosis as assessed by Child-Pugh scores. However, sFGL2 levels are not different between patients with inactive alcoholic cirrhosis and healthy controls [19]. Furthermore, no association of sFGL2 levels with the stage of fibrosis and grade of steatosis were observed in patients with non-alcoholic fatty liver disease [16], indicating that increased sFGL2 levels are due to HBV and HCV infections rather than to the occurrence of cirrhosis [19]. FGL2 is over-expressed both at mRNA and protein levels in liver tissue from patients with more severe CHB [27]. A previous study with a small sample size has shown that sFGL2 levels are higher in patients with HCC or LC compared to CHB patients, and that sFGL2 levels are increased in HCC patients with cirrhosis compared to those without cirrhosis [14]. Hepatic stellate cells express and secrete sFGL2, indicating that higher sFGL2 levels observed in LC patients are due to the production of sFGL2 by activated hepatic stellate cells in the cirrhotic liver [14]. Therefore, FGL2 plays a vital role during acute immune responses against HBV and HCV and is involved in the pathogenesis of the infections, particularly in the development of HBV-related cirrhosis.

Fibrin deposition and liver necrosis are decreased in FGL2-deficient mice infected with MHV-3 while the survival rate is increased, implying a crucial role of FGL2 in the pathogenesis of HBV infection [27]. FGL2 is involved in the immune responses against HBV infection as peritoneal macrophages from FGL2-deficient mice infected with MHV-3 lack a procoagulant response [27]. The CD3^+^CD4^−^CD8^−^ double-negative T cells appear to contribute to the pathogenesis and clinical outcome of MHV-3-induced fulminant viral hepatitis via the immunoactivity of FGL2 in a mouse model [29]. The CD4^+^CD25^+^ regulatory T cells (Tregs) and their effector molecule FGL2 play a key role in susceptibility to HBV infection and in regulating the outcome of fulminant viral hepatitis in vivo [11]. Consistently, in a murine model of acute viral hepatitis caused by LCMV-WE, maturation of dendritic cells (DCs) and increased CD8^+^ and CD4^+^ T cells producing IFN-γ have been observed in FGL2 knock-out mice infected with LCMV-WE, demonstrating a crucial role of FGL2 in the immune response against hepatitis viruses [18].

FGL2 is also involved in the pathogenesis of chronic viral infection through regulation of the FcγRIIB/RIII immunosuppressive pathway, indicating that FGL2 might be a therapeutic target for chronic viral infection [30]. FGL2 together with C5aR and TNF-α contribute to coagulation and complement activation during MHV-3-induced fulminant hepatitis [31, 32]. FGL2 has been shown to induce fibrinogen deposition and procoagulant activity, which are commonly observed during liver injury [27, 31]. Clinically, sFGL2 levels are correlated with distinct clinical parameters of HBV infection (e.g. AST, albumin and, platelet counts and HBV-DNA loads) as observed in the present study and in HCV patients [19]. FGL2, as an effector of Treg cells, contributes to the inhibition of cellular immune responses (induced either by HBV or HCV) against virus replication. The above statement corroborate our findings that sFLG2 levels were thus positively correlated with viral loads and subsequently resulting in the unfavourable clinical outcome such as increased liver enzymes. Hence, sFGL2 may be an indicator of and/or mediator for liver damage and progression of viral hepatitis and a potential target for intervention in fulminant and chronic hepatitis [11, 15, 18, 31].

FGL2 is not only highly expressed on the surface of macrophages, endothelial and dendritic cells, but also in solid tumors including HCC [25, 33, 34]. In line with previous studies [33, 34], up-regulation of *FGL2* mRNA was observed in tumor tissues, compared to directly adjacent non-tumor tissues. Higher FGL2 expression was observed in programmed cell death protein 1 (PD-1)-deficient mice infected with MHV-3 compared to wildtype animals and FGL2 up-regulation is mediated by IL2, IFN-γ and TNF-α [34, 35]. This indicates that FGL2 is involved in controlling the immunopathological damage through PD-1 signaling, which is associated with various types of cancer [35]. Also, mFGL-2 appears to promote angiogenesis and tumorigenesis through the FGF-2/ERK (fibroblast growth factor-2/extracellular signal-regulated kinases) signaling pathway, but not by thrombin-mediated mechanism [36]. Importantly, FGL2 is over-expressed in colorectal carcinoma (CRC) and clear cell renal cell carcinoma (ccRCC) tumors compared to non-tumor tissues [25, 37], and the expression levels are associated with cell proliferation and invasion in vitro and with CRC progression and metastasis in vivo [37]. These findings are supported by our observation that plasma sFGL2 levels are increased in patients with HCC compared to those without HCC and healthy controls. Either FGL2 knockdown or intratumoral injection of an artificial microRNA targeting hFGL2 leads to a delayed proliferation of tumor cells and an inhibition of tumor growth and angiogenesis as well as improves survival in vivo [33, 38, 39]. In addition, increased FGL2 expression is significantly associated with poor prognosis in patients with ccRCC [25]. Therefore, sFGL2 and mFGL2 play an essential role in the tumorgenesis of HCC and may be considered a promising indicator for HCC prognosis and a potential target for HCC therapy. However, more studies are needed to evaluate the clinical potential of sFGL2 as well as the association of *FGL2* mRNA expression with sFGL2 and mFGL2 in HCC.

## Conclusions

sFGL2 levels are significantly induced by HBV infection and associated with the progression and clinical outcome of HBV-related liver diseases. sFGL2 levels may be an additional biomarker for monitoring progression and treatment of the diseases. The *FGL2* gene and corresponding proteins (mFGL2 and sFGL2) may be a target for therapeutic intervention of HBV-related liver diseases.

## Abbreviations

AHB: Acute hepatitis B
CHB: Chronic hepatitis B
HBV: Hepatitis B virus
HC: Healthy control
HCC: Hepatocellular carcinoma
LC: Liver cirrhosis
sFGL2: Fibrinogen-like protein 2
